# Effects of polygalacturonase overexpression on pectin distribution in the elongation zones of roots under aluminium stress

**DOI:** 10.1093/aobpla/plac003

**Published:** 2022-02-23

**Authors:** Teruki Nagayama, Akane Tatsumi, Atsuko Nakamura, Naoki Yamaji, Shinobu Satoh, Jun Furukawa, Hiroaki Iwai

**Affiliations:** 1 Faculty of Life and Environmental Sciences, University of Tsukuba, Tsukuba, Ibaraki 305-8572, Japan; 2 Research Institute for Bioresources, Okayama University, Chuo, Kurashiki 710-0046, Japan

**Keywords:** Aluminium, pectin, polygalacturonase, rice (*Oryza sativa*), root elongation zone

## Abstract

The roots of many plant species contain large amounts of pectin and it contributes to the formation of the rhizosphere. In the present study, the relationship between the root-tip pectin content and aluminium (Al) tolerance in wild-type (WT) and demethylesterified pectin degradation enzyme gene overexpressor (*OsPG2*-FOX) rice lines was compared. *OsPG2*-FOX rice showed reduced pectin content in roots, even under control conditions; Al treatment reduced root elongation and the pectin content in the root elongation zone. Wild-type rice showed more pectin accumulation in the root elongation zone after Al treatment. Relative to WT rice, *OsPG2*-FOX rice showed more Al accumulation in the root elongation zone. These results indicate that the amount of pectin influences Al tolerance and that the distribution of pectin in the root elongation zone inhibits Al accumulation in rice roots. Pectin accumulation in cell walls in the root elongation zone may play a role in protecting rice plants from the Al-induced inhibition of root elongation by regulating pectin distribution.

## Teaser

Aluminium (Al) toxicity has long been known to damage plant cells and inhibits the growth of plants. The roots of many plant species contain large amounts of pectin and it contributes to the formation of the rhizosphere. Pectin degradation enzyme gene overexpressor rice allowed the authors to piece together how rice plant cells and cell wall pectin respond to Al. The authors found that pectin accumulation in cell walls in the root elongation zone may play a role in protecting rice plants from the Al-induced inhibition of root elongation by regulating pectin distribution.

## Introduction

Aluminium (Al) toxicity damages plant cells and inhibits plant growth. Under acidic conditions, Al dissolves into its ionic form, which is toxic to plants (Foy 1988). Aluminium toxicity has become a leading cause of low crop yield worldwide (Von Uexküll and Mutert 1995). Aluminium compounds are affected by soil pH (Duan and Gregory 2003). In acidic (pH < 5) soils, Al is leached in the form of the water-soluble Al^3+^ ion, which is one of the strongest inhibitors of plant growth when absorbed (Famoso *et al.* 2010). Growth inhibition by Al has been reported in various plants, including rice, wheat, maize, tomato and *Arabidopsis*. The mechanism underlying Al toxicity is complex, and the way in which Al inhibits root elongation is not well understood (Kochian *et al.* 2005). However, most Al-related events are caused by the binding of Al to extracellular and intracellular materials due to its high affinity for oxygen donor compounds. Reportedly, Al^3+^ interacts with the negatively charged surfaces of plant roots and inhibits nutrient uptake in acidic soils (Lu *et al.* 2020). Most of the Al that inhibits root elongation is localized in the epidermal and outer cell walls (Jones *et al.* 2006).

The cell wall is thought to play a role in the mechanism of Al sensitivity. For example, silicon treatment improved Al tolerance by decreasing pectin methylesterase (PME) enzyme activity, hemicellulose content and Al accumulation in rice cell walls (Xiao *et al.* 2021). Furthermore, Chang *et al.* (1999) reported that the localization of Al in the cell wall correlated with the amount of pectin in cultured tobacco cells. Chemically, cell wall pectin is a mixture of heterogeneously branched polysaccharides (Ridley *et al.* 2001). The major pectic polysaccharide is homogalacturonan, which is a linear α-1,4-linked d-galacturonic acid partially methylesterified at the C_6_ atom (Mohnen 2008). Pectin polysaccharides are synthesized in the Golgi apparatus (Driouich *et al.* 1993; Baydoun *et al.* 2001), and a significant proportion of homogalacturonan is secreted in methylesterified form (Li *et al.* 1997, 2002; Lennon and Lord 2000). In our previous study, we used a mutant strain of rice [the *sensitive to aluminium rhizotoxicity 1* (*star1*) strain] that is very sensitive to Al toxicity and shows poor root elongation when Al is present in the soil. Nagayama *et al.* (2019) reported that this Al sensitivity of *star1* was associated with pectin deficiency and that the distribution of pectin in the root tips, particularly in root border cells, played a significant role in conferring Al tolerance.

Several studies have been performed to investigate the localization of Al in the cell walls in relation to the contents of pectin (Chang *et al.* 1999) and hemicellulose (Yang *et al.* 2011; Zhu *et al.* 2017a, b; Xu *et al.* 2018). Some plant species have developed mechanisms to manage internal and external Al toxicity (Ma *et al.* 2001; Ryan *et al.* 2001; Rengel 2004; Kochian *et al.* 2005). Some plants are Al-tolerant, and researchers have proposed several mechanisms underlying this tolerance. For example, buckwheat has been shown to transport absorbed Al to the above-ground parts of the plant, where it is chelated to oxalic acid and accumulates in vacuoles (Ma *et al.* 1998; Chen *et al.* 2017). Although the separation of Al into vacuoles was also reported in barley (Liu *et al.* 2020), the best-known mechanism of Al tolerance is the secretion of organic acid anions from plant roots (Li *et al.* 2000; Ma *et al.* 2001; Ryan *et al.* 2001; Kochian *et al.* 2005; Hoekenga *et al.* 2006; Furukawa *et al.* 2007; Kopittke *et al.* 2017; Awasthi *et al.* 2019; Qiu *et al.* 2019). For example, Al-tolerant *Brachiaria decumbens* secreted 3–30 times fewer organic acids than Al-sensitive species such as maize and wheat (Arroyave *et al.* 2018). Al-tolerant plants tend to accumulate less Al than do Al-sensitive plants (Ma *et al.* 2001; Furlan *et al.* 2018). Furthermore, cell walls have been shown to readily adsorb and bind Al (Van *et al.* 1994).

Rice is a major global crop with relatively high Al tolerance (Famoso *et al.* 2010). However, the mechanism of Al tolerance in rice is not well understood. Reportedly, the Al tolerance of rye, which is comparable to rice, is attributable to the secretion of organic acids from the root tips (Li *et al.* 2000). Citric acid secretion increases with the Al concentration in rice, with no significant difference between Al-tolerant (Koshihikari) and Al-sensitive (Kasalath) cultivars (Ma *et al.* 2002). In addition, the expression of FRDL4, a citric acid transporter, and the exudation of citric acid from the roots are enhanced in indica rice with high Al tolerance (Awasthi *et al.* 2019). However, whether increased citrate secretion contributes to Al tolerance is not completely understood because the expression of ART1 and ALS1, which are involved in other Al tolerance mechanisms, was also increased in this plant (Larsen *et al.* 2007; Yamaji *et al.* 2009). Furthermore, no significant reduction in Al tolerance was observed in mutant rice with low organic acid secretion (Yokosho *et al.* 2016), indicating that such secretion is not significantly involved the Al tolerance of rice. Reduced pectin levels in Al-sensitive mutants have been reported, but whether pectin contributes directly to Al tolerance remains unclear (Nagayama *et al.* 2019). In the present study, we investigated the relationship between root-tip pectin distribution and Al tolerance in wild-type (WT) and demethylesterified pectin degradation enzyme gene overexpressor rice of *OsPG1* and *OsPG2* lines.

We previously generated *OsPG2*-FOX rice lines with minimal pectin due to overexpression of the gene encoding polygalacturonase [OsPG1 (Os03g0124900) and OsPG2 (Os01g0517500)], a pectin-degrading enzyme. *OsPG1* and *OsPG2* overexpression decreased the pectin content in the leaf (Ohara *et al*. 2021). The Al concentrations used were higher than those in acidic soils, but similar to those used in previous rice Al toxicity studies. The results showed the distribution of pectin affected Al tolerance, and that Al accumulation in the root elongation zone contributed to the Al tolerance of rice.

## Materials and Methods

### Plant and growth conditions

Wild-type (*Oryza sativa*, cv. Nipponbare), *OsPG1*-FOX and *OsPG2*-FOX (Ohara *et al*. 2021) rice strains were used in the experiments (Huang *et al*. 2009). The rice seedlings were immersed in ion-exchange water at 30 °C for 3 days and then grown in 1.0 CaCl_2_ at pH 4.5 for 3 days. The grown seedlings were exposed to Al with 1.0 mM CaCl_2_ and 0 or 100 µM AlCl_3_, pH 4.5 water culture media for 1 day. The free Al activity was evaluated using GEOCHEM-EZ software (Yoshida *et al*. 1972; Shaff *et al*. 2010), and it ranged from 76.57 to 78.28 %. The plants were grown at 30 °C under continuous light of 250 μmol m^−2^ s^−1^.

### Measurement of root elongation

Root length was measured with a ruler before and after Al treatment for the quantification of root elongation during Al treatment. The percentage of relative root elongation (RRE) was calculated to compare root elongation and Al tolerance between the rice lines using the following equation: RRE = root growth under each Al condition/root growth in control × 100.

### Saponification of pectin

To remove the methyl groups from methylesterified pectin, creating demethylesterified pectin, the methylesterified pectin in roots was saponified with 0.1 N NaOH in 50-mL centrifuge tubes for 1 min. After saponification, the roots were washed with ion exchange water and then all pectin was stained using ruthenium red (Iwai *et al.* 1999).

### Ruthenium red staining of demethylesterified pectin

To detect the demethylesterified pectin in roots, sample roots were stained with 0.01 % (w/w) ruthenium red in 50-mL centrifuge tubes for 5 min. After staining, the roots were washed with ion exchange water (Iwai *et al.* 1999).

### Eriochrome cyanine R staining of Al

Aluminium in roots was stained with 0.1 % (w/w) eriochrome cyanine R in 50-mL centrifuge tubes for 20 min. After staining, the roots were washed with ion exchange water (Jones and Thurman 1957).

### Cell wall collection

Root tips (0–1 mm) from three seedlings were cut with a razor and collected in 2.0-mL tubes as cell wall samples. The samples were frozen in liquid nitrogen and crushed with a pestle.

A methanol/chloroform mixture (1 mL, 1:1, v/v) was added to the samples, followed by centrifugation at 15 000 rpm for 5 min and supernatant removal; the process was repeated twice. After the last supernatant removal, the samples were air-dried (Sumiyoshi *et al.* 2013).

### Determination of uronic acid

Uronic acid was determined using the method of Blumenkrantz and Gustav (1973). Briefly, 1 mL ion exchange water was added to each sample, and 1 mL iced concentrated sulfuric acid (0.025 M borax) was mixed into 200 µL of each sample. After heating in 100 °C water for 10 min and cooling in ice, 40 µL carbazole solution (125 mg carbazole, 100 mL ethanol) was mixed into each sample. The samples were heated in 100 °C water for 15 min, then cooled in ice; absorbance was measured at 530 nm (GENESIS 10S UV-VIS; Thermo Scientific).

## Results

### Al treatment of *OsPG1* and *OsPG2*-FOX rice with low root pectin content inhibited root elongation

**Figure 1.**
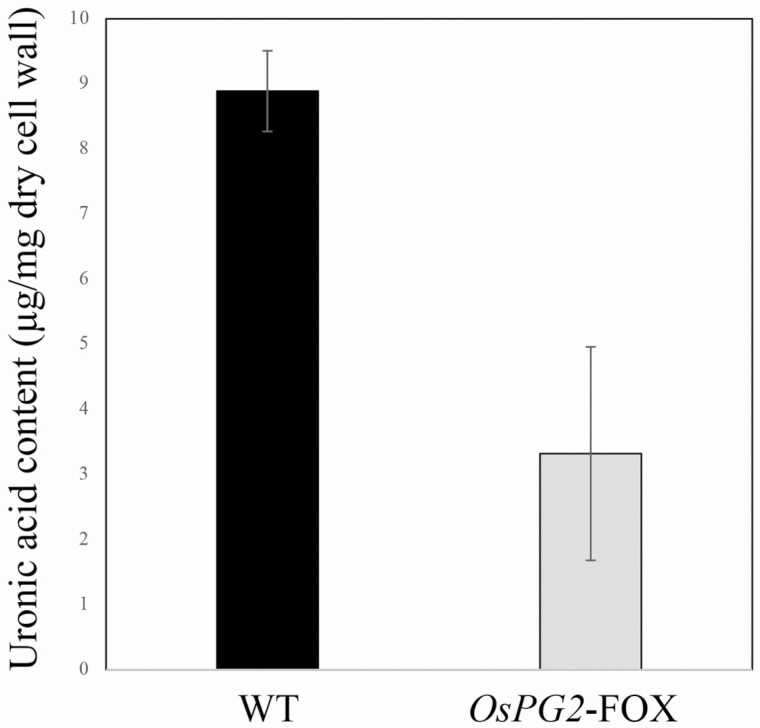
Uronic acid content in cell walls from whole roots of WT (cv. Nipponbare) rice in the mature growth stage. The content differed significantly between WT and *OsPG2*-FOX rice (*P* < 0.01, Student’s *t*-test). Data are means ± SDs, *n* = 7.

**Figure 2.**
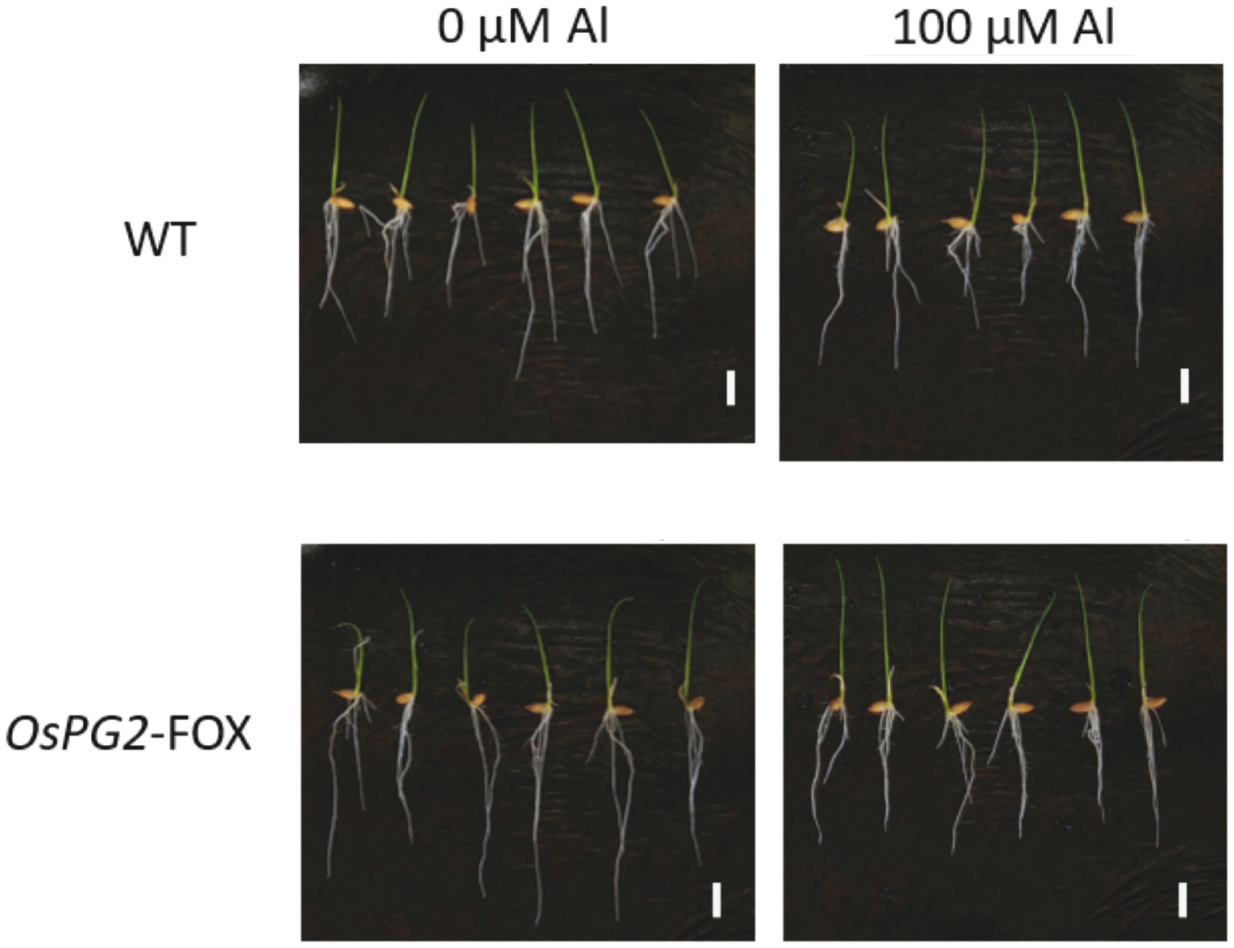
Phenotypes of WT and *OsPG2*-FOX seedlings treated without or with Al (0 or 100 µM AlCl_3_ for 24 h). *OsPG2*-FOX rice showed a high level of root elongation under the 0-µM Al condition. The elongation of roots treated with 100 µM Al was reduced by ~70 % in *OsPG2*-FOX rice compared with the WT. Bars = 1 cm.

**Figure 3.**
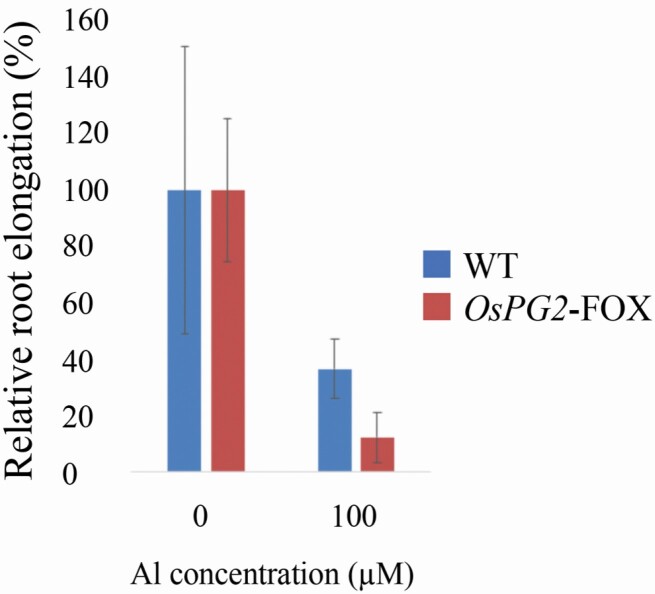
Relative root elongation of WT and *OsPG2*-FOX seedlings grown in 1.0 mM CaCl_2_ at pH 4.5 and then treated without or with Al (0 or 100 µM AlCl_3_). Seedling root length was measured before and after Al treatment, and the amount of root elongation was determined. Root elongation differed significantly between WT and *OsPG2*-FOX rice under 100-µM Al treatment (*P* < 0.01, Student’s *t*-test). Data are means ± SDs, *n* = 12.

**Figure 4.**
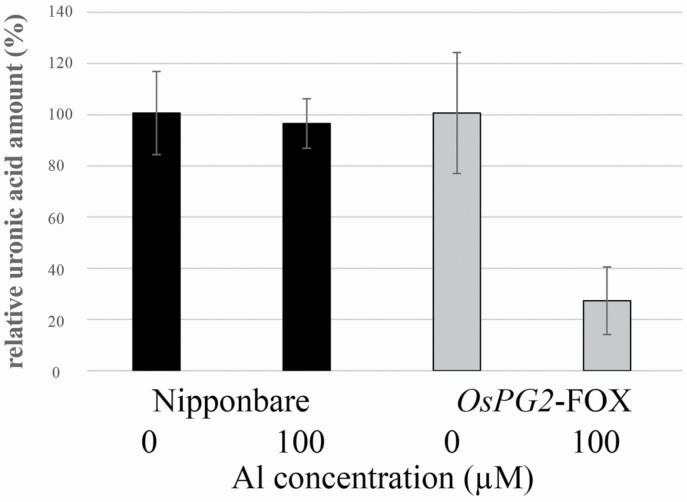
Uronic acid content in cell walls in the root tips (0–1 mm) of WT and *OsPG2*-FOX seedlings grown in 1.0 mM CaCl_2_ at pH 4.5 and then treated without or with Al (0 or 100 µM AlCl_3_) for 24 h. Relative uronic acid amounts normalized to 100 % of the amounts under Al treatment are shown. The uronic acid content differed significantly between WT and *OsPG2*-FOX rice under 100-µM Al treatment (*P* < 0.01, Student’s *t*-test). Data are means ± SDs, *n* = 3.

Approximately 14 500 FOX rice lines individually overexpress full-length cDNAs in rice, driven by the maize ubiquitin-1 gene promoter, a constitutive and overexpression promoter (Hakata *et al.* 2010). Among these lines, *OsPG1*-FOX and *OsPG2*-FOX have been shown to overexpress the full-length cDNA of *OsPG1* and *OsPG2* (Ohara *et al*. 2021). Relative to WT rice, *OsPG1*-FOX and *OsPG2*-FOX rice shows a significantly increased *OsPG2* gene transcript level in leaves, 5.2- and 4.6-fold more PG activity and 37.1 % and 30.3 % reduction in the content of uronic acid, the main component of pectin (Ohara *et al*. 2021). In the present study, the amount of uronic acid in roots was measured and was reduced to 34.8 % in *OsPG2*-FOX rice (Fig. 1). Root elongation was clearly suppressed under existing Al conditions in *OsPG2*-FOX rice, and the relative elongation rate of roots treated with 100 µM Al was ~55 % lower in *OsPG2*-FOX than in WT rice (*P* < 0.01, *t*-test; Figs 2 and 3). After Al treatment, the uronic acid content in the cell walls was reduced significantly in *OsPG2*-FOX rice (Fig. 4); no similar reduction was observed in the WT rice. *OsPG1*-FOX rice showed very similar to *OsPG2*-FOX, this line was also reduced to 39.6 % in the amount of uronic acid in roots **[see****Supporting Information—Fig. S1A****]** and the relative elongation rate of roots under Al was ~54 % lower in *OsPG1*-FOX than in WT rice **[see****Supporting Information—Fig. S1B****]**. Since the phenotype of reduced pectin in the root cell wall was observed more stably in OsPG2-FOX than in *OsPG1-FOX*, we used *OsPG2-FOX* for other experiments in this study.

### Distribution of pectin *OsPG2*-FOX rice roots after Al treatment

**Figure 5.**
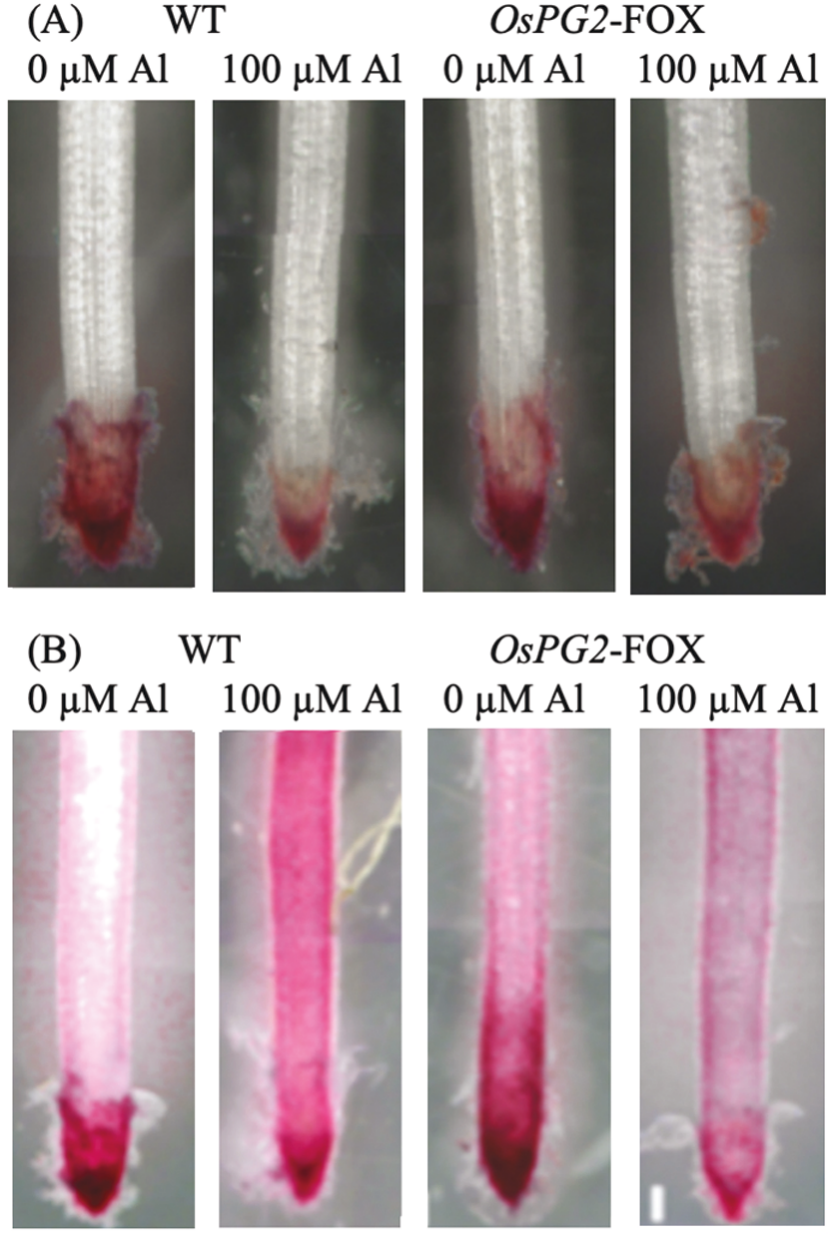
Demethylesterified pectin content, based on ruthenium red staining without (A) and after (B) saponification (0.1 N NaOH, 1 min), in the roots of WT and *OsPG2*-FOX seedlings treated without or with Al (0 or 100 µM AlCl_3_). Roots were stained with 0.01 % ruthenium red for 5 min. Bar = 0.1 mm.

After Al treatment, the roots were stained with ruthenium red and the presence of demethylesterified pectin 0.2 mm from the root tips in WT and *OsPG2*-FOX rice was confirmed (Fig. 5). The distribution of demethylesterified pectin in root tips was similar in WT and *OsPG2*-FOX rice. Conversely, regarding the distribution of total (methylesterified and demethylesterified) pectin, a high signal level was observed in the lateral root elongation zone > 1 mm from the tips in WT rice under Al conditions, whereas the signal level in the root elongation zone was lower in *OsPG2*-FOX rice (Fig. 5). These results reflect a high degree of methylesterified pectin accumulation in Al-treated WT roots. On the other hand, the calcium concentration in the cell wall was very similar between WT and OsPG2-FOX under both normal and Al stress conditions **[see****Supporting Information—Fig. S2****]**.

### Distribution of Al in *OsPG2*-FOX rice roots after Al treatment

**Figure 6.**
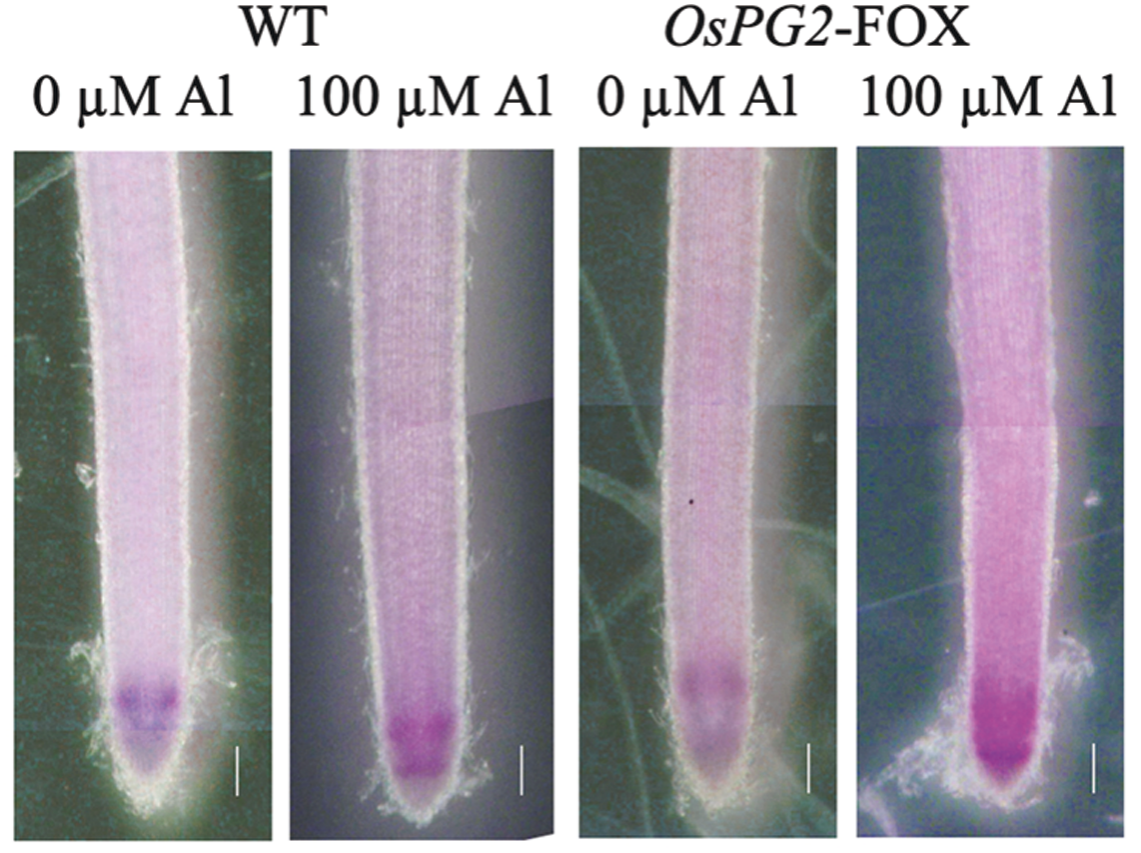
Aluminium (Al) content, based on eriochrome cyanine R staining, in the roots of WT and *OsPG2*-FOX seedlings treated without or with Al (0 or 100 µM AlCl_3_) for 24 h. Roots were stained with 0.1 % eriochrome cyanine R for 20 min. Bars = 0.1 mm.

Wild-type and *OsPG2*-FOX rice roots were stained with eriochrome cyanin R after Al treatment; the staining was nearly the same as that without Al treatment in the WT rice, whereas strong staining was observed in the root elongation zone in *OsPG2*-FOX rice (Fig. 6). The quantification of Al content revealed ~60 % more Al accumulation in *OsPG2*-FOX than in WT rice **[see****Supporting Information—Fig. S2****]**.

## Discussion

The root tip is a major site of Al toxicity in higher plants (Ryan *et al.* 1993), and the mucilaginous capsule surrounding the root tip has been considered to be a source of protective substances that prevent Al uptake into the root meristem (Horst *et al.* 1982). The causes of Al damage to plant cells reportedly include Al binding to phospholipids in the cell membrane, effects on other ion concentration gradients in the cell, increased amounts of reactive oxygen species and Al accumulation in the cell wall (Matsumoto *et al.* 1992; Horst *et al.* 2010; Awasthi *et al.* 2019; Nagayama *et al.* 2019; Kar *et al.* 2020). The root cell wall has been suggested to be a site of Al toxicity and Al exclusion (Horst *et al.* 2010). Up to 90 % of Al absorbed by roots is localized in the apoplast (Kochian 1995), and the main site of Al^3+^ binding is the pectin matrix, which is composed primarily of galacturonic acid homopolymers (Mohnen 2008; Horst *et al.* 2010). Furthermore, Furlan *et al.* (2020) reported that high Al accumulation in root apoplast seems to be the basis of Al tolerance. During plant development, the biosynthesis and assembly of cell wall pectin occurs, and pectin secreted into the apoplast is highly esterified and later de-esterified by PME, inducing pectin–Ca cross-linking, which plays an important role in the cell wall. Al^3+^ binds to pectin significantly more strongly than does Ca^2+^, and Ca^2+^ binding to the cell wall is necessary for proper cell wall function (Franco *et al.* 2002). In addition, Al treatment was shown to increase the pectin content in pumpkin roots (Van *et al.* 1994), indicating that pectin is involved in the stress response of rice to Al (Nagayama *et al.* 2019). The distribution of pectin in response to Al and its possible effect on Al tolerance in the root elongation zone have not been investigated. In the present study, the hypothesis that the root elongation zone and pectin produced in it are involved in the detection and avoidance of Al toxicity was investigated using pectinolytic enzyme-overexpressing rice plants with reduced pectin content.

*OsPG2*-FOX rice leaves contain little pectin; this study showed that the pectin content is also low in the roots of this plant. Because no difference in growth and development was observed between *OsPG2*-FOX and WT rice, this amount pectin content reduction may not affect root development. Conversely, under Al conditions, *OsPG2*-FOX rice was more strongly affected by the Al-induced inhibition of root elongation than was WT rice (Figs 2 and 3). This finding indicates that *OsPG2*-FOX rice is less Al-tolerant than is WT rice. The results also indicate that pectin in the root cell walls contributes to Al tolerance in rice.

Type II cell walls of monocotyledonous plants, including rice, contain minimal pectin (Yokoyama and Nishitani 2004), and dicotyledonous plants with type I cell walls are rich in pectin (Yang *et al.* 2011). Consequently, proving that a high pectin content alone enhances Al tolerance in rice is difficult. Thus, rather than focusing on the amount of demethylesterified pectin, we investigated the amount of methylesterified pectin, as well as its distribution and function. The distribution of methylesterified pectin in the root elongation zone was associated closely with the Al concentration in the medium (Figs 4 and 5). The calcium concentration in the cell wall was very similar between WT and *OsPG2-*FOX **[see****Supporting Information—Fig. S2****]**, probably due to the presence of large amounts of Ca-bound pectin in the meristem region under all conditions (Fig. 5). A significant decrease in uronic acid in the cell wall was observed only in *OsPG2*-FOX rice after Al treatment. Beyond the usual low pectin content in cell walls, the strong Al inhibition of *OsPG2*-FOX rice reduced the amount of pectin accumulated in root cell walls.

Aluminium staining results showed that more Al accumulated in the roots of *OsPG2*-FOX rice than in WT rice after treatment with 100 µM Al (Fig. 6). This result is consistent with a previous report of Al accumulation in the apical root region, where root cell division and elongation are considered to be active (Takehisa *et al.* 2012). In addition, the reduction in root elongation caused by Al is reportedly due to the inhibition of cell elongation, rather than cell division (Ma *et al.* 2004). The reduction of the pectin content in cell walls may have led to increased Al accumulation in the roots. The authors hypothesized that the decrease in pectin allows Al to bind more readily to hemicellulose, leading to increased Al accumulation in roots. In addition to pectin, hemicellulose is a cell wall component that binds to Al (Yang *et al.* 2011; Zhu *et al.* 2017a, b). Hemicellulose is one of the polysaccharides that constitute the cell wall (Lampugnani *et al.* 2018). It interacts with cellulose fibres via hydrogen bonds, covalently with hemicellulose on adjacent cellulose fibres and with pectin polymers. Hemicellulose polysaccharides, classified as xyloglucans, xylans, β-(1-3),(1-4)-glucans, calloses and arabinogalactans, play an important role in cell elongation through cell wall loosening. The primary cell walls of monocotyledonous plants, including rice, contain 30–70 % hemicellulose (Scheller and Ulvskov 2010). Potentially, more Al accumulates in the root cell walls in *OsPG2*-FOX rice than in WT rice under Al treatment conditions **[see****Supporting Information—Fig. S2****]**. In addition, hemicellulose has been found to preferentially adsorb Al over pectin (Yang *et al.* 2011; Zhu *et al.* 2017a). These findings, in combination with our study results (Fig. 6), suggest that pectin protects rice roots from Al-induced elongation inhibition by preventing Al accumulation in the hemicellulose component of the root cell walls and contributing to cell wall elongation.

In our Al staining analysis, regions adsorbing Al did not overlap with regions containing demethylesterified pectin or total pectin. In WT plants containing large amounts of demethylesterified pectin, minimal Al accumulation was observed in Al-treated roots (Fig. 6). Conversely, in *OsPG2*-FOX rice, Al accumulation was observed throughout the root crown and root elongation region (Fig. 6). These results suggest that the methylesterified pectin in the root elongation region of WT plants, which can easily escape into the medium, binds mainly to Al^3+^ like a barrier and only slightly cross-links to Ca^2+^. In addition, Ca showed very similar behaviour in WT and *OsPG2*-FOX rice **[see****Supporting Information—Fig. S1****]**. Because the Ca binding of pectin is irreversible and the binding of Al^3+^ to cell wall pectin via Ca^2+^ replacement is not possible, Al^3+^ may attach to Ca-bound pectin and function as a cation exchange resin. In addition, alkali-soluble pectin in the root elongation region appears to adsorb Al in the initial stage of growth inhibition in pea (Yang *et al.* 2016).

The present results indicate that the root elongation region of *OsPG2*-FOX rice contains little pectin. Thus, hemicellulose, but not pectin, in the *OsPG2*-FOX cell walls accumulates Al. This result is consistent with previous reports that Al-sensitive mutants and cultivars accumulate more Al than do controls (Ma *et al.* 2005). The expression of rice PME is increased in Al-treated roots, and PME overexpression results in increased Al accumulation in roots and decreased Al tolerance in rice (Yang *et al.* 2013). Pectin methylesterase overexpression causes active demethylation of pectin, making it more susceptible to degradation by PG, which decreases root content but promotes exclusion of the root elongation zone and increases Al tolerance via pectin in the root elongation zone. The functioning of this mechanism is thought to decrease with increasing Al accumulation. As Al^3+^ levels increase, nutrient availability decreases, root growth is inhibited, resulting in reduced water and nutrient uptake, and physiological dysfunction (Yang *et al.* 2008; Reis *et al.* 2018).

## Conclusion

The present results indicate that Al accumulation in rice roots is mitigated by the accumulation of pectin in the root elongation zone, and that pectin in the root cell walls contributes to the acquisition of Al tolerance in rice via regulation of the amount and distribution of these components. These Al barriers created by pectin may reduce Al toxicity. In the future, this research could contribute to improved crop production in cultivated areas where plant growth is severely inhibited by Al toxicity.

## Supporting Information

The following additional information is available in the online version of this article—

**Figure S1.** (A) Uronic acid content in cell wall from whole root of WT (cv. Nipponbare) in mature grown stage. Significant difference is shown between WT and *OsPG1-FOX*. Data are means ± SD, *n* = 6. (B) Relative root elongations (RREs) of WT and *OsPG1-FOX* seedlings during Al treatment (1.0 mM CaCl_2_, 0 or 100 μM AlCl_3_, pH 4.5). Root length of seedlings was measured before and after Al treatment and root elongations were calculated. Data are means ± SD, *n* = 12. 

**Figure S2.** Al and Ca content in cell wall from root tips (0–1 mm) of WT and *OsPG2*- FOX seedlings after Al treatment (1.0 mM CaCl_2_, 0 or 100 μM AlCl_3_, pH 4.5) for 24 h.

plac003_suppl_Supplementary_Figures

## Data Availability

No data set was generated in this study.

## Notes

**Phenome, Genome & Environment. Chief Editor**: Colleen Doherty
