# Supplementary material for: Effects of polygalacturonase overexpression on pectin distribution in the elongation zones of roots under aluminium stress
Source: AoB Plants. 2022 Feb 23;14(2):plac003. doi: 10.1093/aobpla/plac003 (PMC8963292; doi:10.1093/aobpla/plac003)
Supplement: plac003_suppl_Supplementary_Figures [file plac003_suppl_supplementary_figures.pdf]

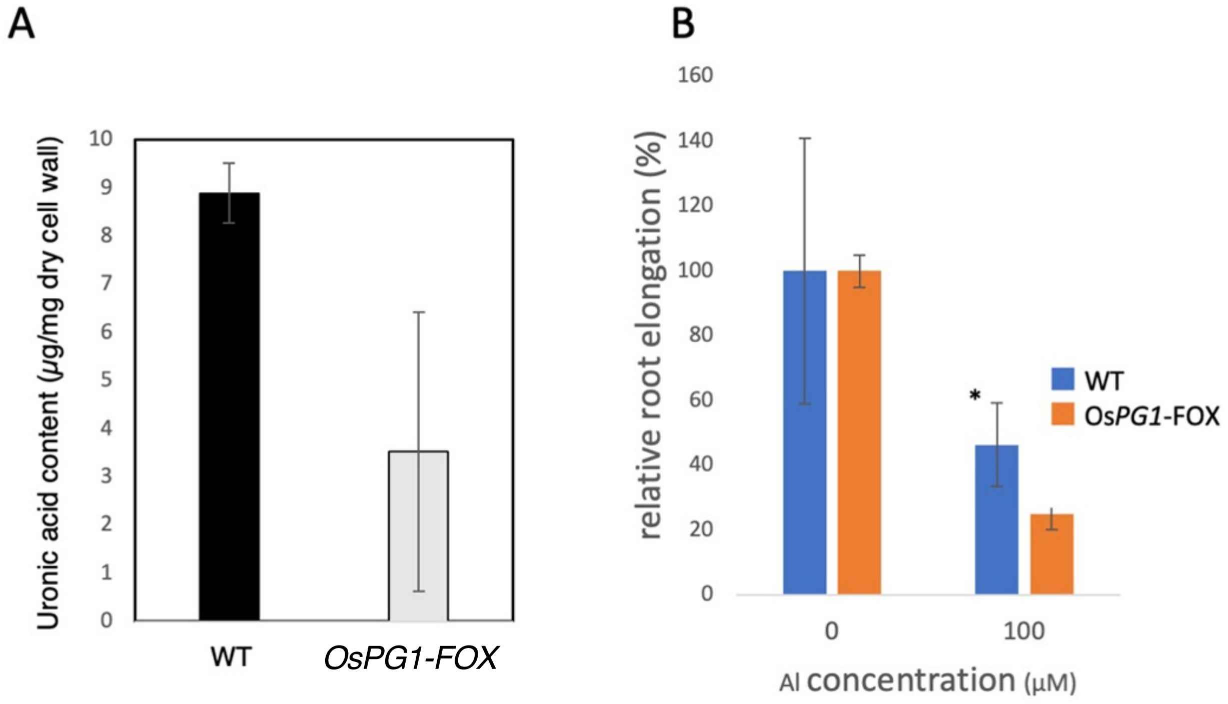

Supplementary Figure 1
(A) Uronic acid content in cell wall from whole root of WT (cv. Nipponbare) in mature grown stage. Significant difference is shown between WT and *OsPG1-FOX*. Data are means ± SD, n = 6. (B) Relative root elongations (RREs) of WT and *OsPG1-FOX* seedlings during Al treatment (1.0 mM $CaCl_2$, 0 or 100 μM $AlCl_3$, pH 4.5). Root length of seedlings were measured before and after Al treatment and root elongations were calculated. Data are means ± SD, n = 12.

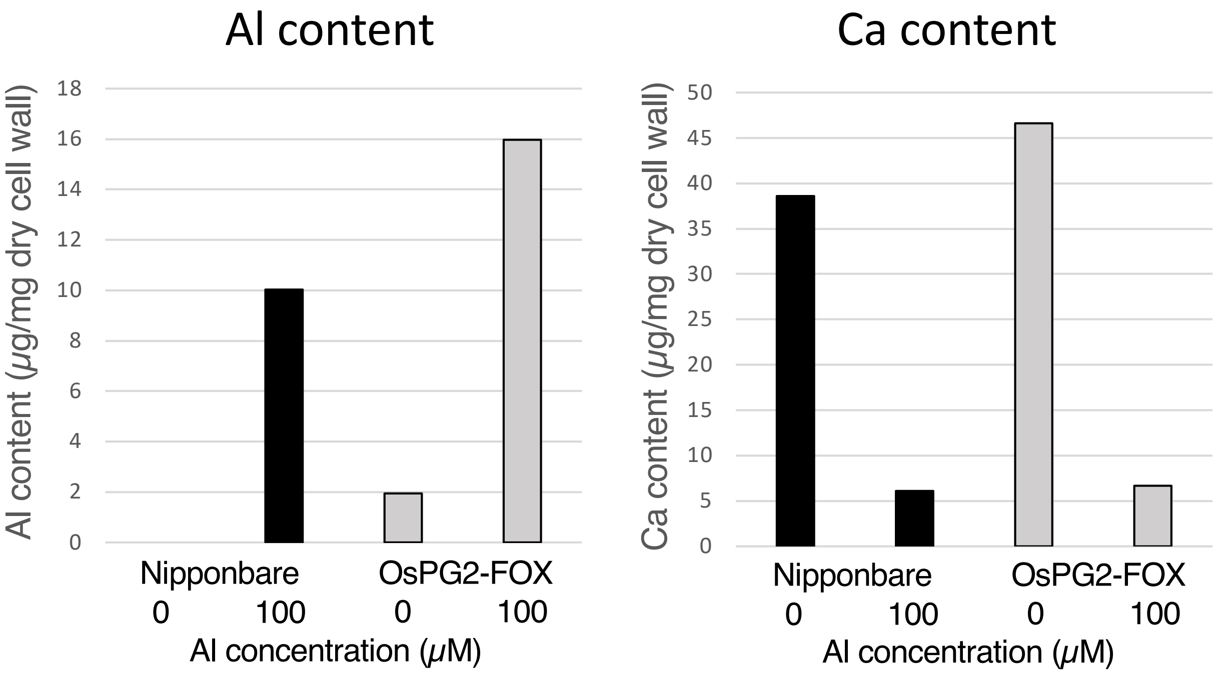

Supplementary Figure 2
Al and Ca content in cell wall from root tips (0–1 mm) of WT and *OsPG2*-FOX seedlings after Al treatment (1.0 mM $CaCl_2$, 0 or 100 μM $AlCl_3$, pH 4.5) for 24 h.
